# Bifidobacteria: insights into the biology of a key microbial group of early life gut microbiota

**DOI:** 10.20517/mrr.2021.02

**Published:** 2021-11-19

**Authors:** Francesca Turroni, Douwe van Sinderen, Marco Ventura

**Affiliations:** ^1^Laboratory of Probiogenomics, Department of Chemistry, Life Sciences and Environmental Sustainability, University of Parma, Parma 43126, Italy.; ^2^Microbiome Research Hub, University of Parma, Parma 43126, Italy.; ^3^School of Microbiology & APC Microbiome Ireland, University College Cork, Cork Co. Cork, Ireland.

**Keywords:** Infant gut microbiota, microbiome, probiotics

## Abstract

The establishment and development of the human gut microbiota constitutes a dynamic and non-random process, which involves positive and negative interactions between key microbial taxa and their host. Remarkably, these early life microbiota-host communications include key events with long-term health consequences. Bifidobacteria arguably represent the most emblematic microbial taxon of the infant gut microbiota. In this context, the interactions among bifidobacteria, their human host, and other members of the human gut microbiota are far from completely understood, despite the crucial role they play in the development and maintenance of human physiology and immune system. Here, we highlight the ecological as well as genetic and functional features of bifidobacteria residing in the human gut using genomic and ecology-based information.

## BIFIDOBACTERIA: WHAT ARE THEY?

The genus *Bifidobacterium* belongs to the Actinobacteria phylum, being phylogenetically positioned close to the ancestral node from which all members of this phylum have evolved^[1]^. The number of microbial taxa ascribed to the *Bifidobacterium* genus currently (September 2021) amounts to 98 (sub)species, with this number steadily increasing over the last five years thanks to the very considerable efforts made by various microbiologists through the assessment of bifidobacterial communities from a wide variety of animals and through the use of metagenomics-based approaches^[2-5]^. The phylogenetic analysis of this genus, as based on genomic data, has highlighted the occurrence of genetic variability that allows subdivision of members of the *Bifidobacterium* genus into seven main phylogenetic/phylogenomic clusters. These latter clusters were assigned names based on one of the species they include, i.e., the *Bifidobacterium bifidum* cluster, the *Bifidobacterium longum* cluster, the *Bifidobacterium pseudolongum* cluster, the *Bifidobacterium adolescentis* cluster, the *Bifidobacterium pullorum* cluster, the *Bifidobacterium asteroides* cluster, and the *Bifidobacterium boum* cluster^[6]^. Notably, the observed phylogenetic heterogeneity and associated clustering of the *Bifidobacterium* genus is also supported by phenotypic/physiological differences, which suggest that bifidobacteria underwent genetic adaptations to different ecological niches within the mammalian gastrointestinal tract.

## BIFIDOBACTERIA: WHERE CAN WE FIND THEM?

Bifidobacteria were originally observed in infant stool samples by Tissier at the beginning of the last century around 1911s, and since then essentially all currently identified (sub)species of bifidobacteria have been isolated from fecal or biopsy samples originated from a wide variety of mammalian species, as well as various birds and social insects. These rather distant ecological niches appear to be linked by the fact that all animals (hosts) from which bifidobacteria have been isolated subject their newborns to parental care. This intriguing ecological situation of a very close relationship between bifidobacteria and their hosts supports the notion of a microbe-host co-evolution scenario.

Recently, with the advent of metagenomics approaches, insights have been obtained concerning the so-called “dark matter” of microbial populations residing in an environment and being represented by bacteria that are detected by sequencing but which appear to be recalcitrant to cultivation. The use of such novel approaches to disentangle bifidobacterial communities that reside in stool samples of all main representatives of mammalian species not only revealed that bifidobacterial presence is a common feature of the mammalian gut but also that a substantial part of the biodiversity of this bacterial genus is still far from being fully discovered^[2]^. In fact, the existence of many novel bifidobacterial phylotypes has been discovered, present in the gut of various mammals including the human gut, some of which occurring at very low abundance, although being shared by various mammalian species^[2]^. Notably, in this context, the application of shotgun metagenomics, followed by metabolic reconstruction involving those biological samples that were shown to encompass putative novel bifidobacterial species, allowed the delineation of detailed metabolic insights concerning the utilization of plant-derived complex carbon sources by these novel bifidobacterial phylotypes. The integration of the discovered plant carbohydrates in the growth media allowed the isolation and subsequent characterization of these novel bifidobacterial taxa, i.e., *Bifidobacterium callimiconis*, *Bifidobacterium goeldii*, *Bifidobacterium colobi*, *Bifidobacterium santillanense*, and *Bifidobacterium amazonense*^[3,4,7,8]^.

## THE HUMAN GUT AND THE ORIGIN OF BIFIDOBACTERIA

Bifidobacteria are highly abundant within the neonatal gut microbiota starting from birth until weaning, while their occurrence in the human large intestine declines with aging. Although bifidobacteria represent dominant members of the gut microbiota in the very early stages of life, their colonization trajectory is affected by peri- and post-natal factors such as delivery mode (cesarian delivery *vs*. vaginal delivery) and/or feeding method (e.g., formula feeding instead of breast feeding), the geographical origin (urbanized *vs*. countryside region), family members, host interactions, exposure to antibiotics, and full-term *vs*. pre-term delivery^[9]^. In this context, there is robust scientific evidence demonstrating that inheritance of bifidobacteria occurs by vertical transmission from mother to her newborn^[10-13]^. Recently, detailed cataloging of bifidobacterial communities residing in the gut microbiota of mothers and their corresponding babies highlighted the occurrence of identical bifidobacterial strains shared by mother-newborn dyads^[10,12,14,15]^. If this bacterial transmission route is interrupted, for example as a consequence of delivery by cesarian section, the gut microbiota of the newborn has been shown to be delayed in becoming dominated by bifidobacteria. Remarkably, but perhaps not surprisingly, it has been demonstrated that mothers and their corresponding newborns not only share identical bifidobacterial strains but also identical bifidobacteriophages, i.e., phages capable of infecting members of the *Bifidobacterium* genus^[16]^. This substantiates a wider vertical transmission trend involving both bifidobacterial cells and their bacteriophages^[11,17]^.

Recently, the importance of human milk as a source of bifidobacteria has been inferred in terms of their impact on bifidobacterial communities in the very early stages of human life^[18,19]^. Human milk possesses its own microbiota, i.e., the human milk microbiota, and bifidobacteria are frequently part of this microbial community. The origin of the human milk microbiota remains uncertain and somewhat controversial. It has been supposed that its bacterial constituents may originate from the female gut where gut commensals reach the mammalian gland through their translocation in the blood or by dendritic cells, i.e., the bacterial entero-mammary pathway^[1,9,20]^. Another hypothesis poses that microbial components of the human milk microbiota originate from the baby as a result of regurgitation during the sucking of the mother’s milk; bacteria present in the oral cavity of the neonate are those that have been inherited from the mother during birth^[1,9]^. Human milk is not only considered a crucial contributor of bacterial cells but also an important source of prebiotic compounds, i.e., specific chemical compounds that specifically support the growth of particular bacterial groups, in particular human milk oligosaccharides (HMOs). Currently, there is growing scientific interest in the application of HMOs as natural modulators of the gut human milk microbiota, eliciting prebiotic effects on bifidobacteria and toward very specific bifidobacterial taxa^[1,21,22]^. Considering the significant importance of bifidobacterial communities during early life, particularly as drivers of gut immunity and development, the evolution of a mammalian secretory fluid containing bifidogenic compounds represents compelling evidence for the notion of bifidobacteria-host/human co-evolution^[5]^.

### Genomics of bifidobacteria

**Figure 1:**
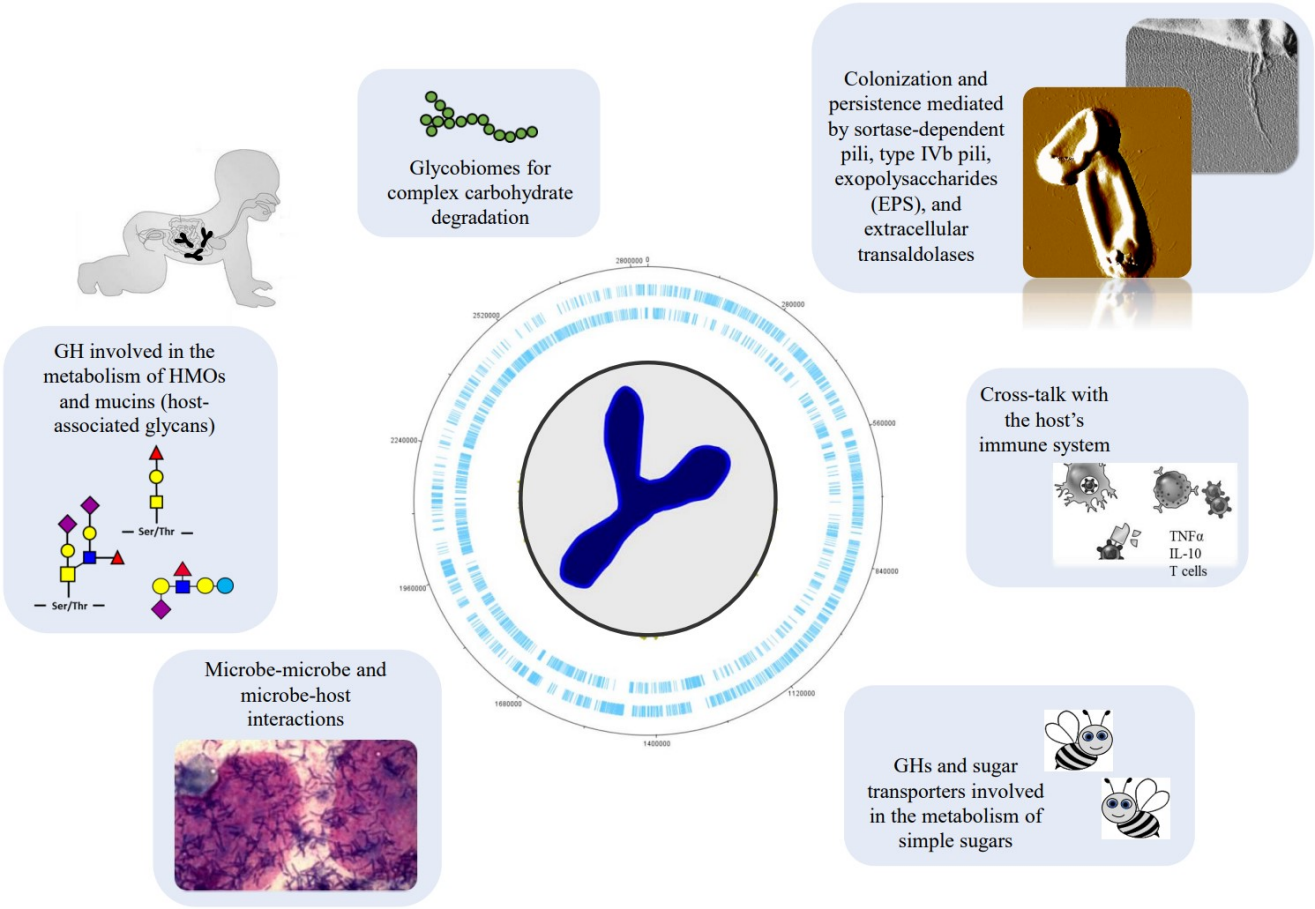
Schematic representation of the key biological features of bifidobacteria.

With the decoding of the *Bifidobacterium longum* subsp. *longum* NCC2705 genome in 2002, the genus *Bifidobacterium* officially entered the microbial genomic era^[23-25]^. Since then, several bifidobacterial genomes belonging to many different taxa have been decoded. In this context, it is worth mentioning the international project entitled Genomic Encyclopedia of Bifidobacteria, which was driven by a consortium of scientists and aimed at decoding the genome sequences of the reference strain for each *Bifidobacterium* (sub)species^[26]^. The results of this project reveal that bifidobacterial genomes, which range in size from 1.35 to 3.25 Mb, are rather small when compared to other members of the Actinobacteria phylum, and that the evolutionary development of the bifidobacterial chromosome is characterized by extensive acquisition of genes, many of which encode glycosyl hydrolases (GH)^[26]^. The genome of the ancestor of the *Bifidobacterium* genus is predicted, based on genome information on currently recognized bifidobacterial species, to contain about 1048 genes. Genetic acquisition events allowed an expansion of bifidobacterial genomes, in particular causing a considerable enlargement of their glycobiomes, i.e., the genetic arsenal encoding functions associated with carbohydrate metabolism^[27]^. This genomic evolution allowed bifidobacteria to become saccharolytic microorganisms with obvious genetic adaptations to degrade and subsequently use carbohydrates typically found in the associated ecological niches^[27,28] ^[Figure 1]. Based on the GH families encoded by the various bifidobacterial genomes, it is possible to identify two main genetic clusters, one particularly enriched in members of the GH13 family and the other in members of the GH43 family, reflecting distinct glycobiomes required for the utilization of a different set of complex plant carbohydrates such as xylan and starch^[27,28]^. Notably, the mammalian gut is highly enriched in these complex carbohydrates, which reflect those that are present in a typical omnivorous diet.

Another finding that supports our view of bifidobacterial genome evolution toward glycan utilization is represented by the predicted glycobiomes of bifidobacterial strains isolated from social insects, in particular the prototypical *Bifidobacterium asteroides* PRL2011, which have been shown to be particularly enriched in GHs and sugar transporters involved in the metabolism of simple sugars typically found in the hindgut of honeybees, from which this bacterium is commonly isolated^[29]^. The scientific literature describes several other interesting examples of metabolic adaptation of bifidobacterial strains to sugars that are particularly abundant in the ecological niches where they live. It has been described how the genomes of bifidobacterial taxa typically present during early life such as *Bifidobacterium bifidum*, *Bifidobacterium breve*, and *Bifidobacterium longum* subsp. *infantis*^[2,4,11,30]^ are also enriched in GH-encoding genes involved in the metabolism of HMOs and mucins, both being host-associated glycans. Various genetic traits involved in HMO breakdown are typically identified in the genomes of members of these three bifidobacterial species, while they are generally absent from chromosomes of other bifidobacterial species, and they further support the notion of highly specialized genetic adaptations of these bifidobacterial species to the infant gut^[27]^. Furthermore, since the chemical structure of HMOs is very similar to that of mucin-associated O-glycans, it has been argued that these infant-associated bifidobacterial species may also persist in the adult human gut.

As mentioned above, in the human gut, bacteria are engaged in a wide variety of trophic interactions aimed at enhancing the utilization of complex carbohydrates by various strains. In this context, it has been shown that *B. bifidum* and *B. breve* cooperate in the utilization of mucin and their generated products. Specifically, sialic acid released by mucin degradation performed by *B. bifidum* PRL2010 cells will be available for *B. breve* UCC2003 cells, thus establishing cross-feeding behavior between these bacteria^[31]^. It is not inconceivable that such cross-feeding activities are frequently established among members of bifidobacterial communities, not only restricted to the human gut but also to other environments inhabited by bifidobacteria^[32]^. In fact, a recent study that explored the composition of the bifidobacterial communities present in fecal samples from the main representatives of mammalian species concluded that bifidobacterial taxa are predominantly engaged in positive interactions, i.e., co-occurrence events, which indicate that, when one bifidobacterial species is present, it enhances the chance of another bifidobacterial species being present^[2]^. In this context, the *B. bifidum* species is frequently associated with an altruistic behavior, which positively impacts the establishment of syntrophic interactions with other members of the bifidobacterial community^[33]^. This phenotype of the *B. bifidum* taxon perfectly aligns with its ecological features that describe this species as one of the most frequently maternally inherited microbial species, which constitutes a so-called bifidotype present in the infant gut^[12]^. The term bifidotype indicates the formation of specific consortia of co‐occurring bifidobacterial taxa^[12]^. Remarkably, thus far, the existence of four main bifidotypes has been described, i.e., *B. bifidum* bifidotype, *B. breve* bifidotype, *B. longum* bifidotype, and *B. adolescentis* bifidotype, where the *B. bifidum* bifidotype is characterized by the dominance of the *B. bifidum* species, eliciting a high co‐occurrence with other bifidobacteria^[12]^.

In the context of the genetic predisposition of certain bifidobacteria towards HMO utilization, it is worth mentioning that only the *B. bifidum* species is fully equipped with an enzymatic machinery for the full degradation of HMOs while *B. longum* subsp. *infantis *can only metabolize HMOs up to a certain size due to restrictions of its uptake machinery. Both *in silico* analyses involving all currently available bifidobacterial genomes and growth experiments based on synthetic media containing (specific) HMOs as the sole carbon source have revealed such intriguing genetic features^[12,18]^. Intriguingly, co‐cultivation of *B. bifidum *with various bifidobacterial strains on HMOs has confirmed cross‐feeding abilities of various bifidobacterial strains^[12]^.

Another important genetic feature of bifidobacteria is represented by the abilities of a relatively small number of taxa to colonize and persist in the human gut^[34]^. Various extracellular structures produced by bifidobacteria have been postulated to be driving such colonization and persistence. These involve sortase-dependent pili, type IVb pili, exopolysaccharides, and extracellular transaldolases^[35,36]^. The structure and biological functions of these cell surface-associated macromolecules have been reviewed in great detail previously (for reviews, see^[34,37,38]^). However, such structures, except for the type IVb pili, are not universally present among members of the *Bifidobacterium* genus and may in addition display heterogeneity within strains of the same species. For example, it has been shown that the number and composition of gene sets required for the biosynthesis of sortase-dependent pili in the genus *Bifidobacterium* are highly variable and may be responsible for interactions with different molecules^[39]^.

Altogether, these findings highlight our knowledge concerning genetic determinants responsible for bifidobacteria-host interactions and interplay between bifidobacterial cells with other members of the gut microbiota is still far from complete.

It has been hypothesized that extracellular structures produced by gut bacteria engage in cross-talk with the host’s immune system, thereby eliciting pro-inflammatory or anti-inflammatory effects, and thus showing that intestinal bacteria can act as natural modulators of the immune system. For bifidobacteria, there is mounting evidence that extracellular structures such as pili/fimbria, exopolysaccharides, and teichoic acids play a pivotal role in the communication between bifidobacteria and the host’s immune system^[38]^, especially during early life, which is characterized by an immature immune system. Remarkably, considering that bifidobacterial dominance is common until weaning, it is tempting to attribute a training role of these bacteria toward the host’s immune system.

## BIFIDOBACTERIA AND THE NEW FRONTIERS OF MICROBIOME-BASED THERAPIES

Historically, bifidobacteria have been exploited by food and pharmaceutical companies as health promoting microorganisms, i.e., probiotic bacteria^[24]^. However, their use as probiotic bacteria is rather restricted to a relatively small number of bifidobacterial species and strains, whose selection was not always based on scientific support for their claimed health benefits. The need for a robust scientific/molecular characterization of health-promoting features led to a microbial genomic discipline called probiogenomics, which could underpin the next generation of probiotic (bifido)bacterial strains. The main aim of probiogenomics is to provide clear insights into the molecular mechanisms responsible for the interaction of the probiotic bacterial strains with the host, as well as with other members of the gut microbiota, and thus ultimately into the resulting health-promoting effects^[24]^. In the context of probiogenomics as applied to bifidobacteria, novel probiotic bifidobacterial strains may become available that are able to modulate the gut microbiota during early life, especially in those newborns who as a consequence of the cesarian delivery or antibiotic treatment may suffer from a disturbed gut microbiota^[9]^. As extensively reported, early life represents a very critical window of time for the foundation of human health with very long-lasting effects. Thus, the establishment of a balanced gut microbiota during infancy could be seen as a very valuable approach to prevent negative health outcomes^[9]^. The intervention during this period with (bifido)bacteria-based therapies may thus be considered a very suitable approach to promote and sustain the generation of such balanced infant gut microbiota.

It is desirable that a similar protocol-based novel generation of probiotic bifidobacterial strains will be applied for other ages or conditions of human life that are often associated with a serious depletion in bifidobacterial cells in the gut, i.e., in elderly and obese individuals, and thus influence host health host through, for example, the application of bifidobacteria to enhance immune therapy strategies^[40-43]^. However, this next generation of probiotic bifidobacterial strains needs to be further investigated through statistically robust clinical interventions (e.g., based on a large cohort of patients, placebo-controlled, and double-blinded). There are many ongoing studies on this crucial topic that are expected to provide crucial insights into the mechanistic roles of a novel generation of probiotic bifidobacterial strains. It is hoped that this information will lead to the establishment of personalized probiotic interventions (use of different bifidobacterial strains with targeted functionalities and based on the gut microbiota composition and genetics of the host), which perfectly aligns with recent developments in personalized medicine.

## CONCLUSIONS

Bifidobacteria represent one of the most prominent microbial bacterial groups in the mammalian gut microbiota during early life, and their key functional roles for mammalian health and well-being are well documented. However, in contrast to other microbial groups, their biology is still largely unexplored. This is partly due to difficulties in their cultivation under *in vitro* conditions, while many bifidobacterial are also highly recalcitrant to genetic manipulation. Despite these methodological limitations, it is highly desirable that investigations pertaining to bifidobacteria continue or even increase in the coming years, and efforts need to be made to overcome these constraints. Notably, the importance of developing novel genetic tools as well as their use to establish engineered bifidobacterial strains for food and biomedical applications, from eliminating antibiotic resistance mobile elements and improving robustness to preventing pathogen infections and delivering therapeutics for cancer treatment, has already been pointed out^[44]^.

Several metagenomic studies focusing on particular diseases, e.g., autoimmune diseases, have identified bifidobacteria as a key microbial biomarker, thereby opening up possibilities for future therapeutic and/or preventive strategies involving bifidobacteria^[45]^. In addition, the administration of bifidobacteria has already been shown to be a valid approach for co-adjuvating immunotherapy approaches for the treatment of melanoma and other forms of cancer^[37]^.

All these findings clearly demonstrate the central role played by bifidobacteria in microbiome research while also highlighting future translational applications as a result of such research. 

## DECLARATIONS

### Copyright

© The Author(s) 2021.
